# Lupus néonatal chez un enfant dont la mère est suivie pour une dermatomyosite

**DOI:** 10.11604/pamj.2018.31.117.11353

**Published:** 2018-10-17

**Authors:** Lamissa Cisse, Yamoussa Karabinta

**Affiliations:** 1 Service de Dermatologie Cnam (Ex Institut Marchoux), Bamako, Mali; 2 Faculté de Médecine et d’Odonstomatologie Usttb, Bamako, Mali

**Keywords:** Lupus, néonatal, dermatomyosite, Lupus, neonatal, dermatomyositis

## Abstract

Neonatal lupus is rare. It is due to the transmission of maternal autoantibodies across the placenta during pregnancy. We here report the case of a 2-month old female infant treated for erythematous macular cutaneous lesions on the face and the trunk. Her mother was followed up for dermatomyositis diagnosed on the basis of clinical lesions, muscle weakness and elevation in muscle enzyme levels. However she had not underwent antinuclear antibody test (ANA). Clinical examination showed atrophic erythematous lesions distributed like butterfly wings on both sides of the nasal pyramid, satellite lesions on the front, with red hair. The remainder of the physical examination was unremarkable. Laboratory tests were not performed because infant's parents also refused the biopsy. These lesions suggested seborrheic dermatitis, rosacea or atopic dermatitis. However, rosacea is very rare in infants and usually affects fair-skinned people. In seborrheic dermatitis, lesions are not atrophic. The age of onset of atopic dermatitis is usually 3 months. Lesions regressed in 15 days under dermocorticoid therapy.

## Image en médecine

Le lupus néonatal est une entité rare qui serait due à la transmission d'auto anticorps maternel à l'enfant pendant la grossesse. Nous rapportons un cas. Il s'agissait d'un enfant de sexe féminin âgé de 2 mois reçu en consultation avec des lésions érythémateuses maculaires du visage et du tronc. La mère de l'enfant était suivie pour une dermatomyosite diagnostiquée sur la base des lésions cliniques, de la faiblesse musculaire et d'une élévation des enzymes musculaires. Toutefois, on ne disposait pas d'un bilan d'auto-immunité chez cette dernière. A l'examen, on notait des lésions érythémateuses atrophiques en aile de papillon de part et d'autre de la pyramide nasale. Des lésions satellites au front, avec des cheveux roux. Le reste de l'examen était sans particularité. Le bilan biologique demandé ne fut pas honoré par les parents qui ont aussi refusé la pratique d'une biopsie. Ces lésions auraient pu faire évoquer une dermite séborrhéique, une rosacé, ou une dermatite atopique. Cependant la rosacé est très rare chez l'enfant, c'est une pathologie de la peau blanche. Dans la dermite séborrhéique, les lésions ne sont pas atrophiques. La dermatite atopique débute habituellement à l'âge de 3 mois. Les lésions ont régressé en 15 jours sous dermocorticoïde.

**Figure 1:**
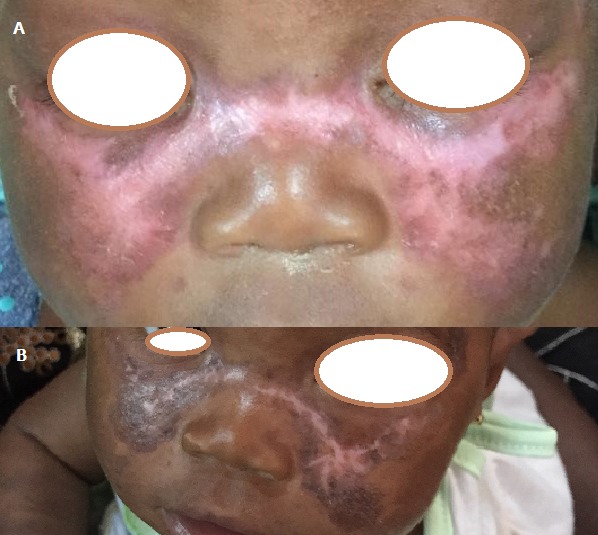
A) lésions erythemateuses vus de face (aile de papillon); B) lésions en voie de cicatrisation

